# Autophagy in *Trypanosoma brucei*: Amino Acid Requirement and Regulation during Different Growth Phases

**DOI:** 10.1371/journal.pone.0093875

**Published:** 2014-04-03

**Authors:** Remo S. Schmidt, Peter Bütikofer

**Affiliations:** 1 Institute of Biochemistry and Molecular Medicine, University of Bern, Bern, Switzerland; 2 Graduate School for Cellular and Biomedical Sciences, University of Bern, Bern, Switzerland; Federal Institute for Vaccines and Biomedicines, Germany

## Abstract

Autophagy in the protozoan parasite, *Trypanosoma brucei*, may be involved in differentiation between different life cycle forms and during growth in culture. We have generated multiple parasite cell lines stably expressing green fluorescent protein- or hemagglutinin-tagged forms of the autophagy marker proteins, TbAtg8.1 and TbAtg8.2, in *T. brucei* procyclic forms to establish a trypanosome system for quick and reliable determination of autophagy under different culture conditions using flow cytometry. We found that starvation-induced autophagy in *T. brucei* can be inhibited by addition of a single amino acid, histidine, to the incubation buffer. In addition, we show that autophagy is induced when parasites enter stationary growth phase in culture and that their capacity to undergo starvation-induced autophagy decreases with increasing cell density.

## Introduction

When eukaryotic cells encounter stress conditions, such as nutrient starvation, they begin to recycle cellular contents using macroautophagy, henceforth referred to as autophagy. During this process, cells engulf cytoplasm containing proteins to be degraded by a double membrane structure, the autophagosome. It originates at the phagophore assembly site and, upon completion, fuses with the lysosome, where the cargo is hydrolyzed together with the inner membrane of the autophagosome (reviewed in [1]). In addition to bulk protein degradation, autophagy can also be directed towards organelles, as in mitophagy (reviewed in [2]), pexophagy (reviewed in [3]) or, as ancient defense mechanism, towards endosomes (xenophagy [4]).

The classical and most widely used marker for autophagy is the modification of the autophagy core protein, Atg8. Processing of Atg8 has been characterized in detail in *Saccharomyces cerevisiae*, where it is essential for autophagy and is mediating autophagosome expansion [5] and membrane tethering and hemifusion *in vitro* [6]. During progression of autophagy, Atg8 is modified more than once. Soon after synthesis [7], Atg8 is cleaved by the cysteine protease, Atg4, to expose a glycine residue at the protein's C-terminus, which upon induction of autophagy becomes covalently modified by phosphatidylethanolamine (PE), catalyzed by Atg7 and Atg3 [7], [8]. This modification changes the biophysical properties of Atg8 leading to altered sub-cellular distribution: the small, hydrophilic, 13.5 kDa protein receives a hydrophobic membrane anchor, causing re-localization from the cytosol to autophagosomes. Using tagged forms of Atg8, this process can be visualized by immunofluorescence microscopy and is reflected by puncta formation [8], [9]. PE-anchored Atg8 remains on the outer leaflet of both the outer and the inner membrane of the autophagosome until hemifusion of autophagosome and lysosome is completed. Finally, the sub-population of Atg8 located on the outer membrane is released by Atg4 [7], whereas the Atg8 on the inner membrane is degraded by lysosomal hydrolases together with the inner membrane [10].

Autophagy represents an ancient mechanism that has been well conserved among eukaryotic cells [11]. It is also present in protozoan parasites [12], i.e. unicellular eukaryotes that have been placed at the bottom of the eukaryotic tree [13]. In parasites, autophagy may be of special importance because of its likely involvement in cell differentiation. Protozoan parasites commonly have complex life cycles during which they encounter vastly different environments, such as a mammal or insect vector. Thus, differentiation between one life cycle form and another represents a crucial event for parasite proliferation and survival. The involvement of autophagy during parasite differentiation has been demonstrated in *Trypanosoma cruzi* [14] and *Leishmania mexicana* [15], suggesting that it may represent a potential target to fight parasite infections [16]. In addition, protozoan parasites have been proposed as model organism to identify a minimal machinery required for autophagy [17]. Initial bioinformatic analyses have shown that the *Trypanosoma brucei* genome may contain only about half of Atg proteins found in *S. cerevisiae* [18]. In a subsequent study, however, the supposedly absent Atg12-Atg5-Atg16-pathway has been identified for syntenic genes in *Leishmania* [19]. Finally, since protozoan parasites branched early during evolution [13], [20], their autophagic machinery may give insight into the evolution of autophagy, which in turn may help elucidate drug targets that may inhibit the process.

*T. brucei*, the model parasite used in this study, is the causative agent of sleeping sickness, or human African trypanosomiasis, a disease that puts an estimated 70 million people at risk of contracting serious health problems [21]. The related animal disease, Nagana, results in the loss of an estimated US$4.75 billion in gross domestic product in Africa [22]. *T. brucei* cycles between cattle or humans, depending on the subspecies, and the insect vector, the tsetse fly [23]. Proliferative forms from the mammalian and the insect stage are grown in culture as bloodstream and procyclic forms, respectively.

In earlier work, autophagy in *T. brucei* has been demonstrated using electron microscopy. As a response to harmful compounds (dihydroxyacetone [24] and spermine [25]), the formation of double-membrane vesicles has been reported. More recently, Atg8 has been used as reporter to monitor autophagy in *T. brucei* procyclic forms [26]. The *T. brucei* genome encodes two genes for Atg8 (TbAtg8.1 and TbAtg8.2), present as tandem genes on chromosome 7. The predicted proteins share a region of very high homology at the C-terminus, but have distinct N-termini. TbAtg8.1 ends in glycine, suggesting that the first processing event involving Atg4 may not be required for its functionality, whereas TbAtg8.2 contains an extra cysteine residue at the C-terminus, requiring cleavage by Atg4 as in other organisms. Processing of TbAtg8.1/8.2 during autophagy has been studied before by expressing tagged forms of the proteins in procyclic trypanosomes. In [26], a fusion protein between yellow fluorescent protein (YFP) and TbAtg8.2 was used to show autophagosome formation. This was corroborated in a more detailed study [27] by expressing fusion proteins between TbAtg8.1 or TbAtg8.2 and YFP or a 10 amino acid tag of *S. cerevisiae* Ty1 virus-like particle (Ty1), respectively. Re-localization from the cytosol to puncta was demonstrated for both tagged proteins using fluorescence microscopy [27]. In addition, TbAtg8 lipidation was shown by protein analysis using SDS-urea polyacrylamide gel electrophoresis and immunoblotting [27]. However, although both methods, i.e. quantifying puncta formation using fluorescence microscopy and gel electrophoresis followed by immunoblotting, represent reliable techniques to follow autophagy, they are laborious and time-consuming and thus, not well suited to analyze larger samples numbers. Recent work in mammalian cells has demonstrated that flow cytometry may represent a method of choice to follow and measure autophagic flux (autophagosome formation and degradation) [28], [29]. Based on the observation that GFP fluorescence is rapidly lost in the hydrolytic and acidic environment of the lysosome [30], [31], GFP-tagged LC3, the mammalian homolog of Atg8, has been used to monitor and quantify degradation of autophagosomal content [28].

In the present study, we establish and validate flow cytometry as a tool to measure autophagy in *T. brucei*. Our results demonstrate that the method can be used to analyze the effects of larger sample numbers, i.e. all proteinogenic amino acids, on autophagic activity and measure autophagy during different growth phases of trypanosomes in culture. Interestingly, we found that that the presence of a single amino acid, histidine, prevents puncta formation and autophagy in *T. brucei* procyclic forms

## Materials and Methods

Unless otherwise stated, all reagents were of analytical grade and purchased from Sigma Aldrich (Buchs, Switzerland) or Merck (Zug, Switzerland). Restriction enzymes were from Fermentas (now Thermo Fisher Scientific, Waltham, MA, USA), and antibiotics were from Sigma Aldrich, Invivogen (San Diego, CA, USA), or Invitrogen (now Life Technologies, Zug, Switzerland). BioMax MS films were from Kodak (now Carestream, Gland, Switzerland) and general purpose blue films were from Carestream (Gland, Switzerland).

### Cell cultures and transfection

*T. brucei* strain Lister 427 procyclic forms were cultured at 27°C in SDM-79 [32] supplemented with 5% (v/v) heat-inactivated fetal bovine serum (FBS; Gibco, Lucerne, Switzerland). For transfection, parasites (4×10^7^ cells) were harvested (1500×*g*, 10 min) at a density of 1×10^7^ cells/mL. Cells were washed once in ZM buffer (132 mM NaCl, 8 mM KCl, 8 mM Na_2_HPO_4_, 1.5 mM KH_2_PO_4_, 0.5 mM magnesium acetate, 0.09 mM calcium diacetate, pH 7.0) and electroporation was carried out with 10-15 μg of linearized plasmid DNA in 500 μL ZM buffer using a BTX electro cell manipulator 600 (Axon Lab, Baden, Switzerland) and one pulse (1.5 kV charging voltage in high voltage mode and 186 Ω resistance) in 0.2 cm Gene Pulser cuvettes (Bio-Rad Laboratories, Cressier, Switzerland). Two hours post transfection, cells were diluted 1∶5 and 1∶25 and plated in 24-well plates (BD Biosciences, Allschwil, Switzerland) to obtain clones. After 24 h of culture, transfectants were selected for antibiotic resistance by addition of 10 μg/mL blasticidin S HCl (for HA-TbAtg8) or 15 μg/mL G418 (for GFP-TbAtg8) to the culture medium.

### Construction of GFP- and HA-tagged TbAtg8.1 and TbAtg8.2

Tagged versions of TbAtg8.1 (Tb927.7.5900) and TbAtg8.2 (Tb927.7.5910) were expressed in *T. brucei* procyclic forms as follows. To tag the proteins with hemagglutinin (HA), the amino acid sequence YPYDVPDYA (see Table S1 for nucleotide sequence) was attached to the N-termini of the genes during PCR. The constructs were then cloned into the *T. brucei* expression vector pCorleone ([33], a kind gift of I. Roditi, University of Bern) between *Hin*dIII and *Bam*HI sites. To tag the proteins with green fluorescent protein (GFP), expression vector pG-EGFP-ΔLIIγ was used ([34], a kind gift of I. Roditi, University of Bern). The vector is a modified version of the pGaprone expression vector [35], containing enhanced GFP (eGFP) with a *Bam*HI site at the 3′ end of eGFP allowing convenient tagging. Both forms of TbAtg8 were amplified by PCR and introduced into the vector at the 3′ end of the eGFP open reading frame using *Bam*HI. The forward primers (see Table S1) used for cloning contained a *Bgl*II restriction site, which is compatible with *Bam*HI and is rendered non-functional after ligation, allowing easy screening for correct orientation of the insert. After ligation and sequencing (Microsynth, Balgach, Switzerland), plasmids were purified using Midiprep kits (Qiagen, Hombrechtikon, Switzerland) and digested with *Not*I and *Sal*I (pCorleone) or *Spe*I (pG-EGFP-ΔLIIγ), respectively. Before transfection, plasmids were purified by phenol-chloroform-precipitation and resuspended in water.

### Fluorescence microscopy

For fluorescence microscopy, 2×10^6^ cells of *T. brucei* procyclic forms were centrifuged for 5 min at 1500×*g*, resuspended in 100 μL phosphate buffered saline (PBS; 137 mM NaCl, 2.7 mM KCl, 10 mM Na_2_HPO_4_, 1.76 mM KH_2_PO_4_, pH 7.4), spread on Superfrost Plus (Thermo Fisher Scientific) microscopy slides and allowed to adhere for 10 min. Parasites were fixed with formaldehyde solution (4% in PBS) for 10 minutes, washed three times for 5 min with cold PBS and air-dried. Cover slides were mounted with Vectashield mounting medium containing 4′,6-diamidino-2-phenylindole (DAPI; Vector Laboratories, Burlingame, CA, USA) and sealed with nail varnish. Fluorescence microscopy was performed using a Leica AF6000 system with a HCX PL APO 100×/1.40 oil-immersion objective. Pictures were acquired with Leica LAS AF software (Version 2.1.0; Leica Microsystems) and processed using ImageJ (Version 1.44o; National Institutes of Health, USA). Puncta were counted either by using acquired z-stacks or by hand, involving a modified telegraph key allowing one-handed counting.

### Immunofluorescence microscopy

For immunofluorescence microscopy, 2×10^6^ cells were allowed to adhere to microscopy slides for 10 min. Parasites were fixed with formaldehyde solution (4% in PBS) for 10 min, washed with cold PBS, and permeabilized with 0.2% (w/v) Triton X-100 in PBS. After blocking with 2% (w/v) bovine serum albumin in PBS for 30 min, the primary antibody in blocking solution was added for 45 min. Antibodies used were mouse monoclonal anti-HA (Covance, Princeton, NJ, USA), mouse monoclonal anti-p67, and rabbit anti-TbCatL (both kind gifts of J.D. Bangs, University of Buffalo) at dilutions of 1∶250, 1∶1000 and 1∶500, respectively. After washing, the corresponding secondary fluorophore-conjugated goat anti-mouse Alexa Fluor 594 or goat anti-rabbit Alexa Fluor 594 (Invitrogen) antibodies were added at dilutions of 1∶1000 in blocking solution for 45 min. After washing and drying, cover slides were mounted and examined by fluorescence microscopy as described above.

### SDS-polyacrylamide gel electrophoresis (SDS-PAGE) and immunoblotting

Whole cell lysates were separated by SDS-PAGE using 10% (for GFP-TbAtg8) or 12% (for HA-TbAtg8) acrylamide gels. For immunoblotting, proteins were transferred onto Immobilon P polyvinylidene difluoride membranes (Millipore, Billerica, MA, USA) by semi-dry blotting for 75 min at 2.5 mA/cm^2^. Mouse monoclonal antibodies against GFP (Roche Diagnostics, Rotkreuz, Switzerland) or HA (Covance) were used at a dilution of 1∶3000. Mouse monoclonal antibody against eukaryotic elongation factor 1A (eEF1A; Upstate, Lake Placid, NY) was used at a dilution of 1∶5000. Primary antibodies were detected using rabbit anti-mouse IgG conjugated to horseradish peroxidase (Dako, Baar, Switzerland), at a dilution of 1∶5000, by enhanced chemiluminescence (Pierce, Lausanne, Switzerland).

### Induction of autophagy by starvation of parasites in gHBSS

To induce autophagy, *T. brucei* procyclic forms were centrifuged at 1500×*g* for 10 min and resuspended in an appropriate volume of pre-warmed gHBSS (137 mM NaCl, 5.4 mM KCl, 0.25 mM Na_2_HPO_4_, 0.44 mM KH_2_PO_4_, 1.3 mM CaCl_2_, 1 mM MgSO_4_, 4.2 mM NaHCO_3_, 1 g/L glucose, pH 7.3; [27]) to a concentration of 5×10^6^ cells/mL. Unless stated otherwise, incubation was carried out for 2 h at 27°C. Autophagy inhibitors chloroquine (Sigma) and bafilomycin A_1_ (Invivogen) were added at indicated concentrations where applicable. Bafilomycin was added to the tube in ethanol and then dried to exclude the vehicle from interfering with measurements. In starvation experiments, amino acids were added to gHBSS from stock solutions in HBSS (Table S2). Glucose concentrations were adjusted where necessary.

### Flow cytometry

After starvation, parasites were diluted to 3×10^5^ cells/mL in cold PBS and immediately analyzed by flow cytometry using a BD FACScan (Becton Dickinson, Allschwil, Switzerland). To assess fluorescence intensity, channel FL1 (530±15 nm) was used. The fluorescence intensity of unchallenged cells was set to a geometrical mean of approximately 10^3^ relative fluorescence units by adjusting the photodiode gain. Non-fluorescent cells were gated out. All analyses were carried out using flow cytometry analysis software FlowJo (Tree Star Inc., Ashland, OR, USA).

## Results

### GFP-TbAtg8.1 and GFP-TbAtg8.2 as markers to monitor autophagy by flow cytometry in T. Brucei

**Figure 1:**
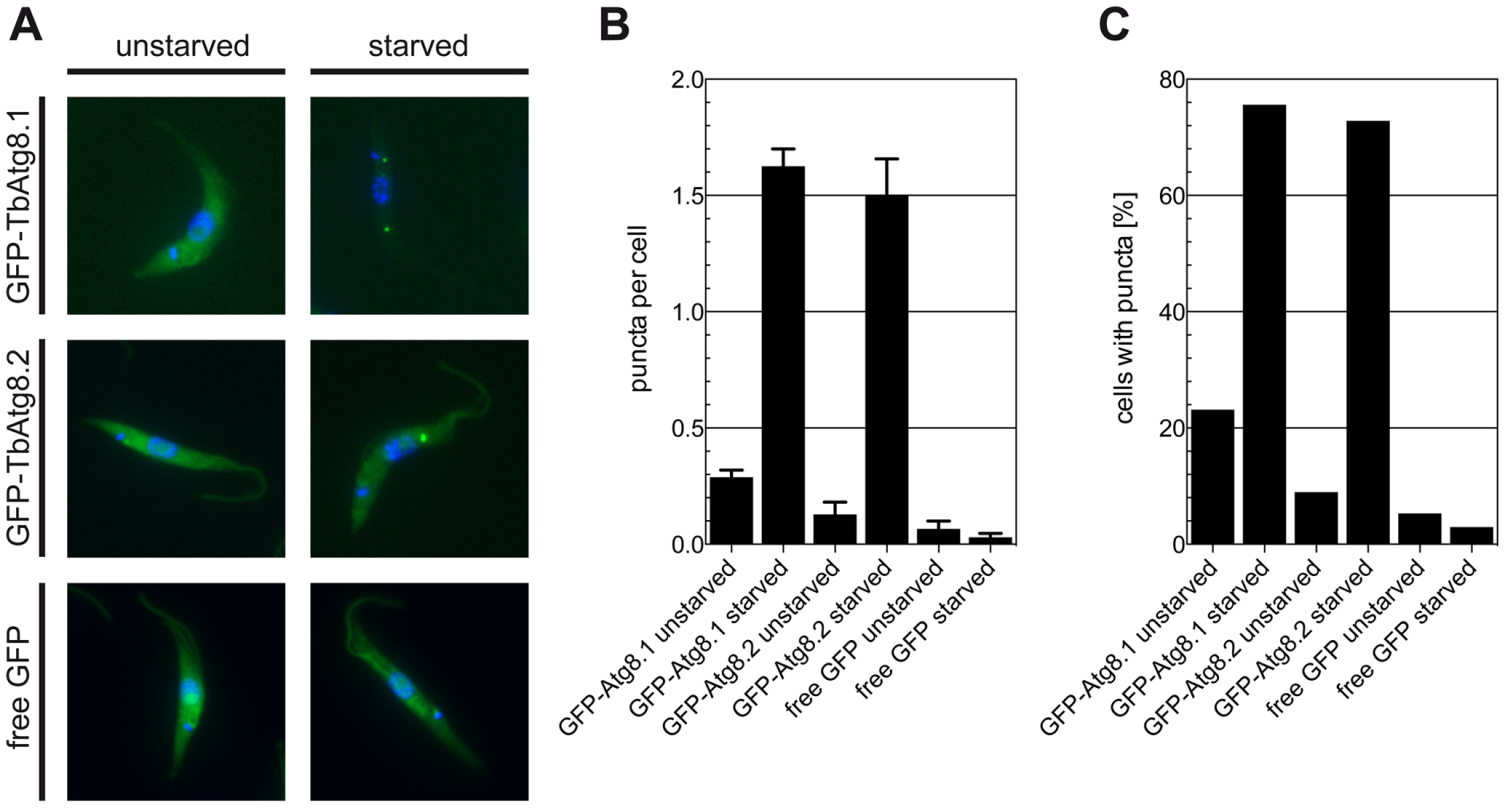
Re-location of GFP-TbAtg8 during starvation. *T. brucei* procyclic forms constitutively expressing GFP-TbAtg8.1, GFP-TbAtg8.2, or free GFP were starved for 2 h in gHBSS. (A) Fluorescence microscopy of representative parasites. (B, C) Fluorescent puncta were analyzed and quantified as puncta per cell (B) and cells containing one or more puncta (C). The numbers of counted cells in (B) were 419, 422, 78, 70, 76 and 101 for individual bars from left to right. The numbers for GFP-TbAtg8.1 cells are from four independent experiments, those for GFP-TbAtg8.2 and free GFP cells from single experiments. Error bars in (B) indicate standard error of the mean.

In a recent report, YFP- and Ty1-tagged forms of TbAtg8.2 constitutively expressed in *T. brucei* procyclic forms were described as suitable markers to monitor autophagy [27]. Metabolic starvation in gHBSS induced a re-location of the tagged proteins from the cytosol to punctate structures, which were counted using fluorescence microscopy. We now show that N-terminally GFP-tagged TbAtg8.1 is similarly suited to monitor autophagy in *T. brucei*. In unchallenged parasites, constitutively expressed GFP-TbAtg8.1 and GFP-TbAtg8.2 localize to the cytosol of *T. brucei* procyclic forms. During starvation in gHBSS, both tagged forms re-located to punctate structures (Fig. 1A). In control cells expressing free GFP, no re-location of the signal was observed (Fig. 1A). Quantification of puncta after starvation for 2 h revealed that the number of puncta per cell as well as the number of cells with one or more puncta increased several fold (Fig. 1B, C).

**Figure 2:**
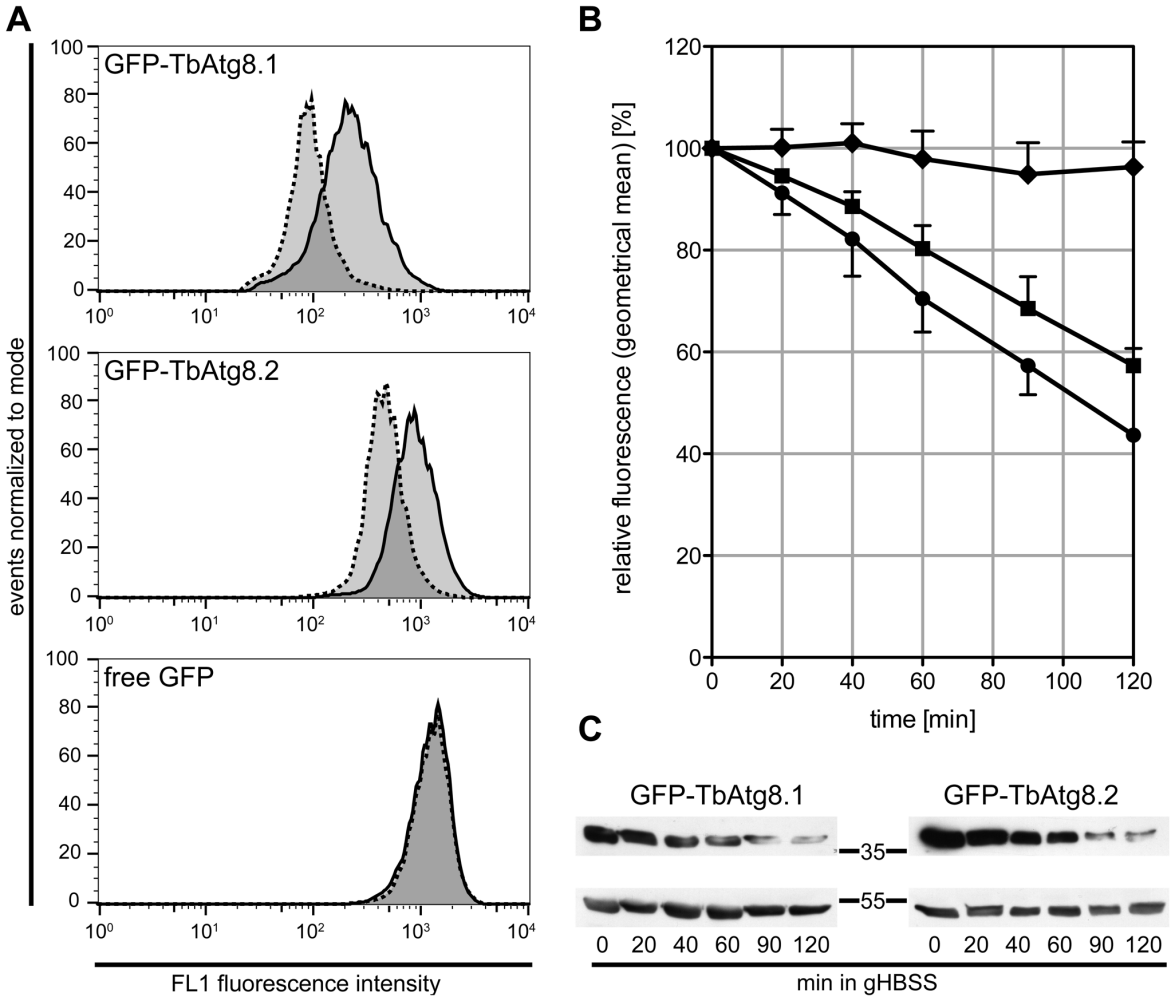
Decrease of GFP-TbAtg8 fluorescence during starvation. *T. brucei* procyclic forms expressing GFP-TbAtg8.1, GFP-TbAtg8.2 or free GFP were starved in gHBSS and analyzed by flow cytometry (A, B) and SDS-PAGE/immunoblotting (C). (A) Flow cytometry of unstarved (solid) and starved (dashed) cells after 2 h of starvation; the lines show signals in the FL1 channel. (B) Geometrical means of GFP signals during starvation (for 0 and 2 h see A) relative to the intensity at time point 0 minutes. Symbols represent GFP-TbAtg8.1 (•), GFP-TbAtg8.2 (▪) and free GFP (♦). Error bars indicate standard deviations from three independent experiments. (C) Immunoblots of parasite lysates during starvation using antibodies against GFP (upper panels), or eukaryotic elongation factor 1A (lower panels) as loading control. For GFP-TbAtg8.1 (left panels), 3×10^7^ parasites were loaded per time point, for GFP-TbAtg8.2 (right panels), 5×10^6^ parasites were loaded. Molecular mass markers are indicated in kDa in the margin.

Microscopic examination and counting of puncta is not only time-consuming but also error-prone as it typically includes a limited number of cells only. Thus, we studied if autophagic flux could also be monitored by flow cytometry. Our results show that the fluorescence intensity of *T. brucei* procyclic forms stably expressing GFP-TbAtg8.1 or GFP-TbAtg8.2 decreased after 2 h of starvation in gHBSS (Fig. 2A, upper and middle panel). In contrast, no change in signal was observed for parasites expressing cytosolic GFP (Fig. 2A, lower panel). Quantification of the signal using the geometrical means of fluorescence intensity demonstrates a continuous decrease in fluorescence intensity during starvation in parasites expressing GFP-TbAtg8.1 or GFP-TbAtg8.2, but not GFP, reaching approximately 50% of the starting fluorescence (Fig. 2B).

In parallel, we analyzed possible changes in the levels of GFP-tagged TbAtg8.1 and TbAtg8.2 using SDS-PAGE and immunoblotting. The results show that the amounts of both proteins progressively decreased during starvation (Fig. 2C). A similar observation has previously been reported for TbAtg8.1 and TbAtg8.2 linked to other tags [27].

**Figure 3:**
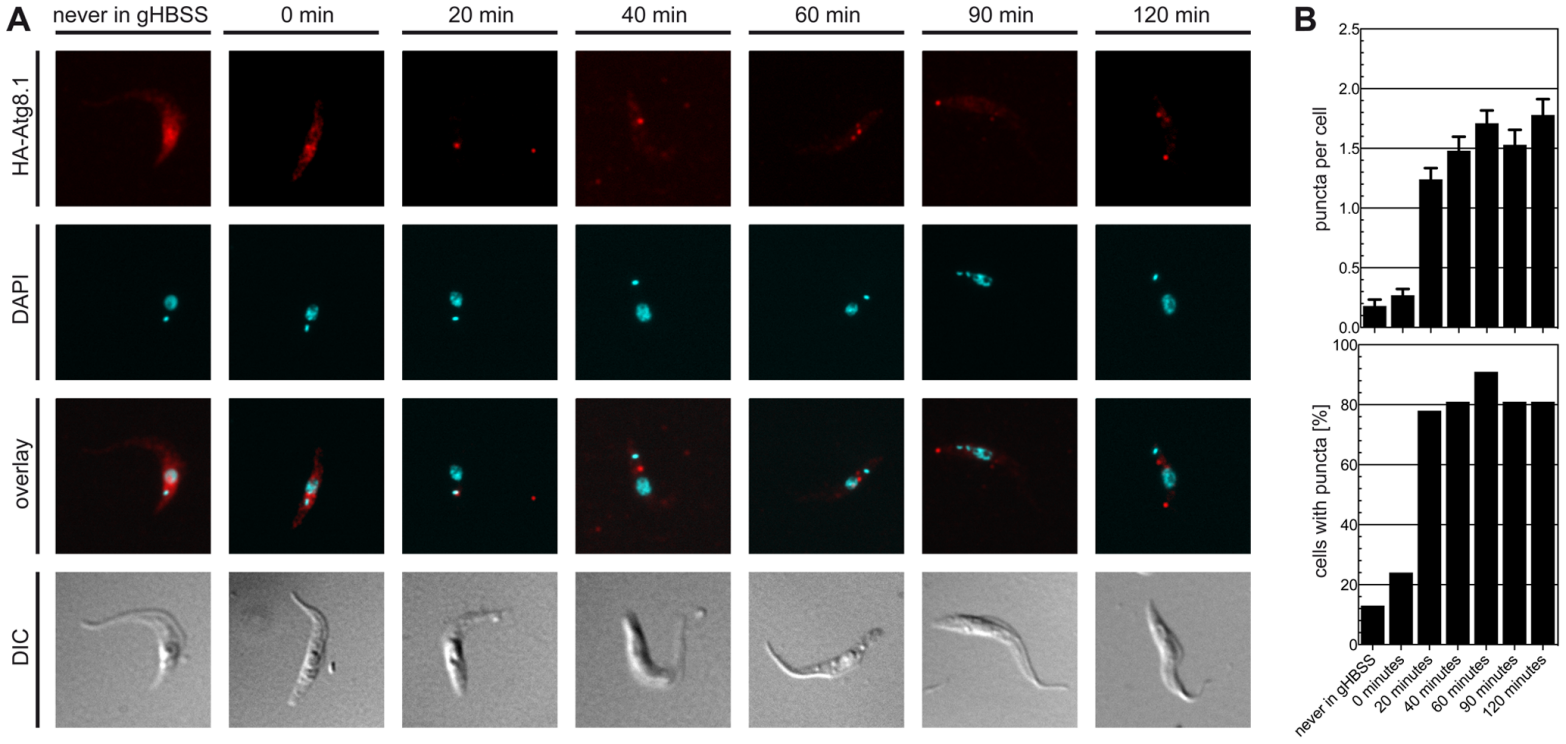
Re-location of HA-TbAtg8 during starvation. *T. brucei* procyclic forms constitutively expressing HA-TbAtg8.1 were starved for indicated times in gHBSS. (A) Sub-cellular localization was visualized by antibodies against the HA epitope. DNA was visualized by DAPI (cyan). DIC, differential interference contrast. (B) At each time point, the numbers of puncta were counted by examining 100 cells (upper panel) and the number of cells containing puncta were determined. Error bars indicate standard error of the mean.

To exclude that the observed effects on GFP-tagged reporter proteins during starvation were related to the relatively large GFP proteins linked to the much smaller TbAtg8 proteins, we also generated *T. brucei* procyclic forms constitutively expressing HA-tagged TbAtg8 reporters. Analysis of HA-TbAtg8.1 by immunofluorescence microscopy showed a similar re-localization of the protein to punctate structures (Fig. 3A). Quantification of puncta (Fig. 3B, upper panel) showed similar results as for GFP-TbAtg8.1 (Fig. 1B,C). Our data using HA-tagged TbAtg8 are also in line with a previous study involving Ty1- and YFP-tagged TbAtg8.1 and TbAtg8.2 [27]. Furthermore, we found that puncta are formed in the first 20–40 minutes of starvation and remain stable during the rest of a 2 h starvation experiment (Fig. 3B, lower panel).

**Figure 4:**
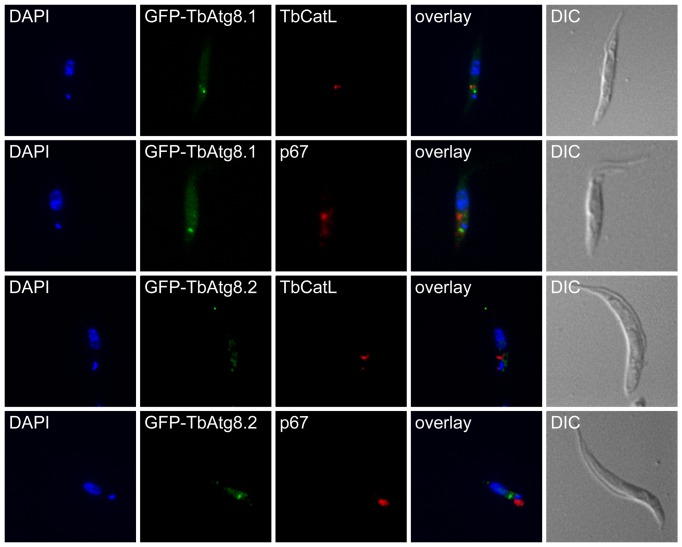
Localization of GFP-TbAtg8 puncta. *T. brucei* procyclic forms expressing GFP-TbAtg8.1 or GFP-TbAtg8.2 were starved for 2 hours in gHBSS. GFP-TbAtg8 is shown in green. Lysosomes were stained with antibodies against p67 or TbCatL and are shown in red. DNA was visualized by DAPI, shown in blue. DIC, differential interference contrast.

Re-localization to punctate structures of GFP- and HA-tagged TbAtg8.1 and TbAtg8.2 and degradation of GFP-tagged forms may resemble autophagosome formation followed by degradation of its contents in lysosomes, as has been reported in mammalian cells and yeast [5], [36]. Thus, we analyzed a possible co-localization of GFP-tagged puncta with the lysosomal marker proteins, p67 [37] and TbCatL (also known as trypanopain [38]), using immunofluorescence microscopy. The results showed no co-localization with p67 or TbCatL (Fig. 4), except in very rare cases (Fig. S1), indicating that the puncta represent stages before autophagosome fusion with the lysosome.

Together, our results demonstrate that constitutively expressed GFP- and HA-tagged forms of TbAtg8.1 and TbAtg8.2 are valuable markers for autophagy in *T. brucei* procyclic forms. In addition, we show that progression of autophagy in parasites expressing GFP-TbAtg8.1 or TbAtg8.2 can be monitored by flow cytometry, providing a simple and rapid assay to monitor autophagy under different experimental culture conditions.

### Processing of GFP-TbAtg8.1 is caused by histidine deprivation

**Figure 5:**
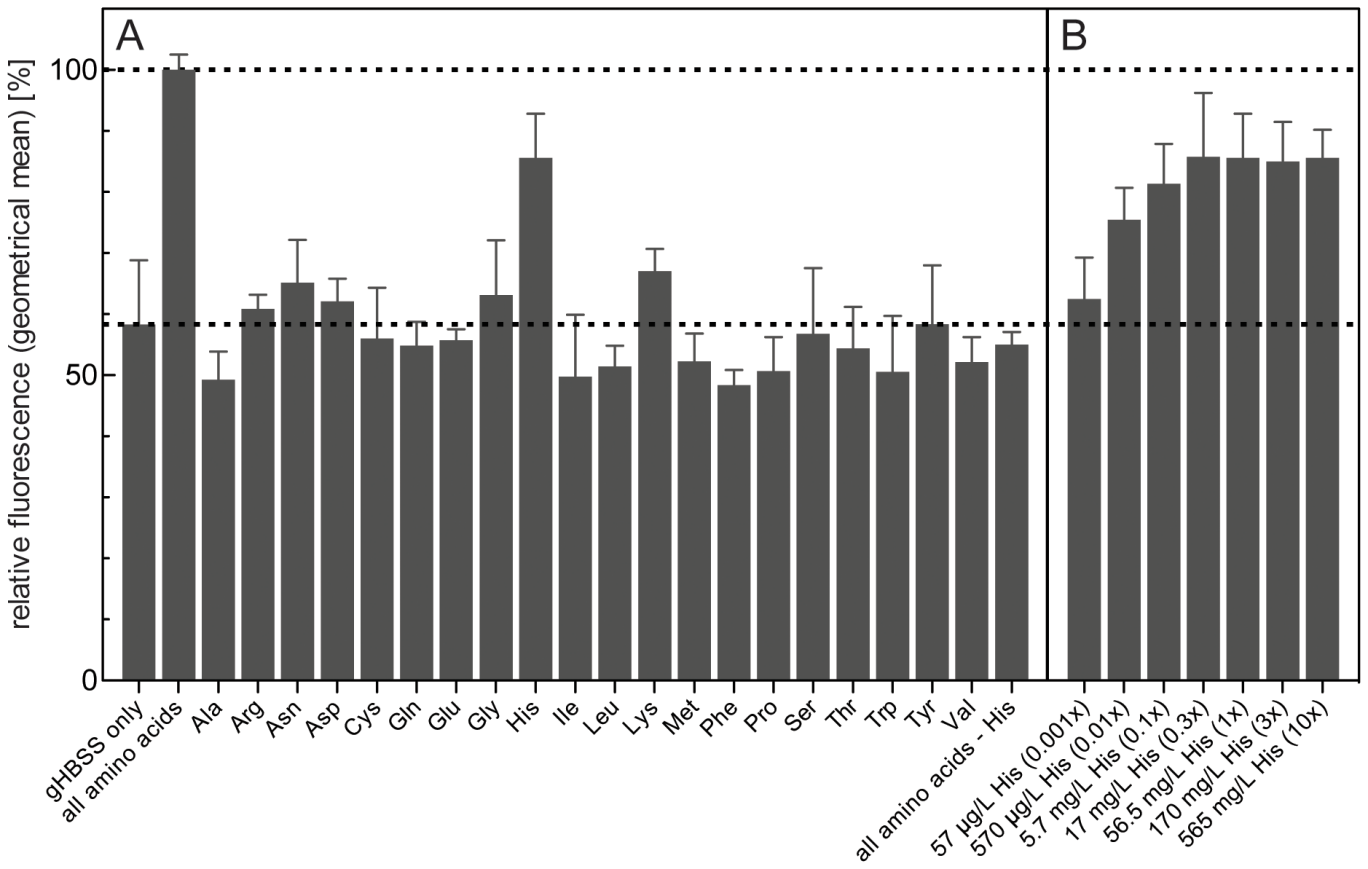
Effect of amino acid supplementation on GFP-TbAtg8.1 fluorescence during starvation. *T. brucei* procyclic forms expressing GFP-TbAtg8.1 were incubated for 2 h in gHBSS supplemented with different amino acids or mixtures of amino acids. (A) GFP-TbAtg8.1 fluorescence was recorded by flow cytometry and expressed relative to the value obtained in the presence of all amino acids (indicated by the dashed line). (B) Dependence of GFP-TbAtg8.1 fluorescence on histidine concentration; × indicates the histidine concentration in the standard *T. brucei* culture medium and the buffer containing all amino acids. The numbers in (A) and (B) are from at least three and two, respectively, independent experiments. Error bars indicate standard deviation.

The work of Li and coworkers [27] showed that puncta formation of YFP-TbAtg8.2 can be inhibited by addition of a mixture of amino acids to gHBSS. In line with their work, we found that the presence of amino acids completely inhibited puncta formation of GFP-TbAtg8.1 (result not shown), which correlated with decreased GFP-TbAtg8.1 fluorescence measured by flow cytometry (Fig. 5A, second column). The availability of a rapid autophagy assay system now allowed us to investigate if prevention of autophagy by amino acids was due to the presence of bulk amino acids in the culture medium, or caused by a subset of amino acids.

**Figure 6:**
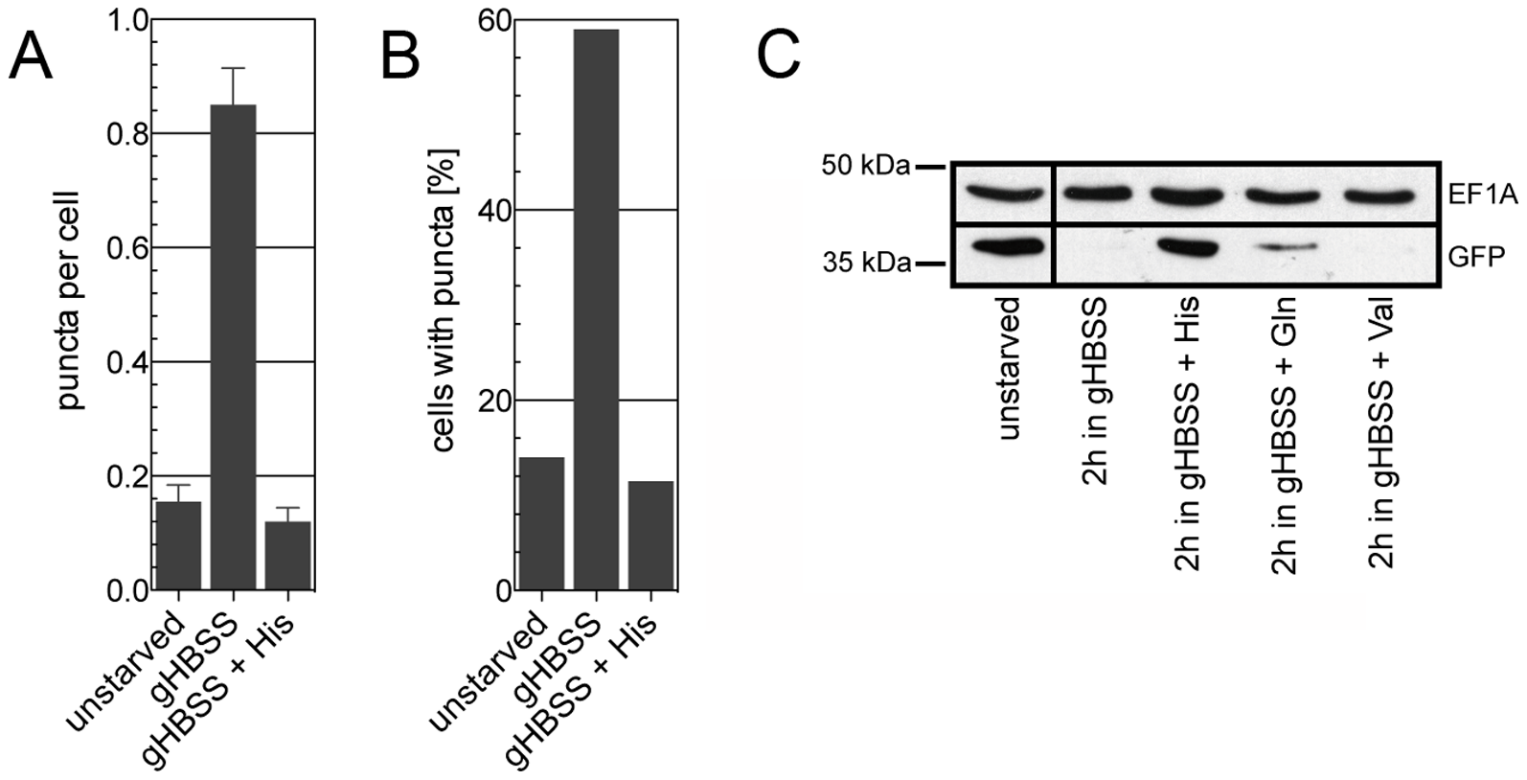
Effect of histidine supplementation on puncta formation and GFP-TbAtg8.1 levels. *T. brucei* procyclic forms expressing GFP-TbAtg8.1 were incubated for 2 h in gHBSS, supplemented with histidine, glutamine or valine as indicated. Fluorescent puncta were analyzed and quantified as puncta per cell (A) and cells containing one or more puncta (B). The results are from two independent experiments, in which at least 100 cells per incubation condition were analyzed. Error bars indicate standard errors of the mean. (C) Immunoblots of parasite lysates obtained after incubation of cells in the absence or presence of amino acids and probed with antibodies against GFP (lower panel), or eukaryotic elongation factor 1A (upper panel) as loading control. Each lane contains 5×10^6^ cell equivalents. Molecular mass markers are indicated in kDa in the margin.

Surprisingly, our results showed that autophagy could be inhibited by the addition of a single amino acid, histidine, to the culture medium. The presence of histidine largely prevented the autophagy-related decrease in fluorescence intensity observed in gHBSS, while the addition of any other amino acid, or a mixture of all amino acids except histidine, had little or no effect (Fig. 5A). The concentrations of amino acids used in the experiment reflect those in the standard *T. brucei* culture medium, SDM-79 [32], to mimic normal culture conditions. The dependence of autophagy on histidine was further demonstrated using different concentrations of the amino acid in the assay system (Fig. 5B). Similar results were obtained when autophagy was monitored by analyzing puncta formation using fluorescence microscopy or GFP-TbAtg8.1 degradation using SDS-PAGE and immunoblotting. After starvation of parasites for 2 h, puncta per cell (Fig. 6A) and cells containing puncta (Fig. 6B) were markedly increased (see also Fig. 1B,C), while the addition of histidine to the incubation buffer completely blocked these events. Similarly, degradation of GFP-TbAtg8.1 was inhibited when trypanosomes were incubated in the presence of histidine (Fig. 6C), confirming the results obtained by flow cytometry (Fig. 5). Our observation that neither bulk amino acids nor proline and threonine, i.e. the two amino acids used most heavily by *T. brucei* procyclic forms for energy production [39], inhibited the loss of fluorescence signal intensity indicate that gHBSS-mediated autophagy in *T. brucei* is not caused by a lack of energy from amino acid metabolism but related to the absence of a single amino acid, histidine.

### Drop of GFP-TbAtg8.1 fluorescence is not affected by autophagy inhibitors

**Figure 7:**
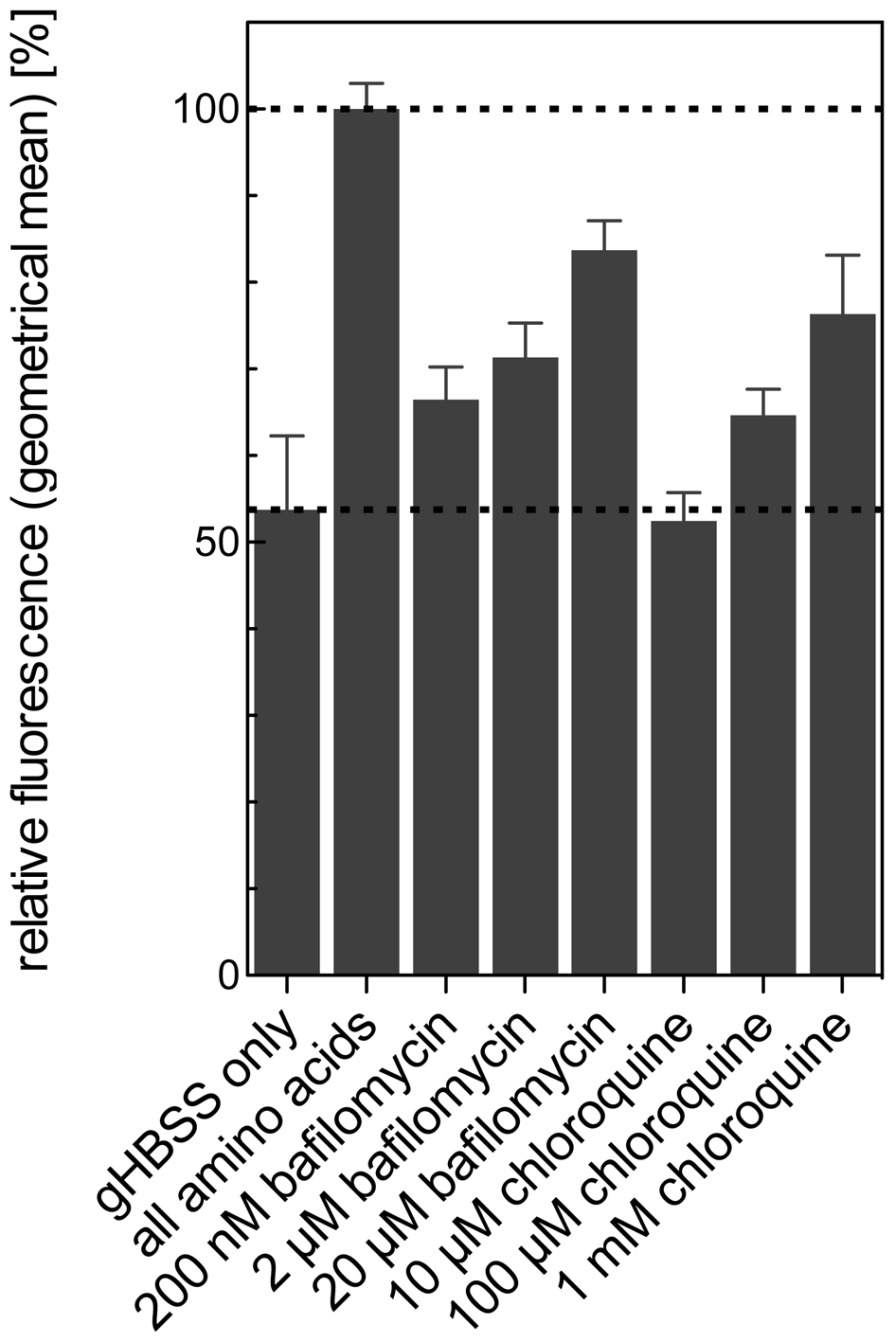
Effects of autophagy inhibitors on GFP-TbAtg8.1 fluorescence. *T. brucei* procyclic forms expressing GFP-TbAtg8.1 were starved for 2 h in gHBSS in the absence or presence of chloroquine and bafilomycin A_1_ at indicated concentrations. The numbers are from three independent experiments. Error bars indicate standard deviations.

Chloroquine and bafilomycin A_1_, both known inhibitors of autophagy [40], are compounds that have been used before in trypanosomes to neutralize the lysosomal pH and inhibit degradation of delivered cargo [38], [41]. To study their effects on autophagy in *T. brucei*, we added chloroquine and bafilomycin A_1_ to *T. brucei* procyclic forms expressing GFP-tagged Atg8.1. The results show that autophagy-mediated decrease in fluorescence intensity was inhibited by both compounds, however, only at concentrations well above those used in previous studies in *T. brucei* (Fig. 7).

### Processing of GFP-TbAtg8.1 is affected by parasite density

**Figure 8:**
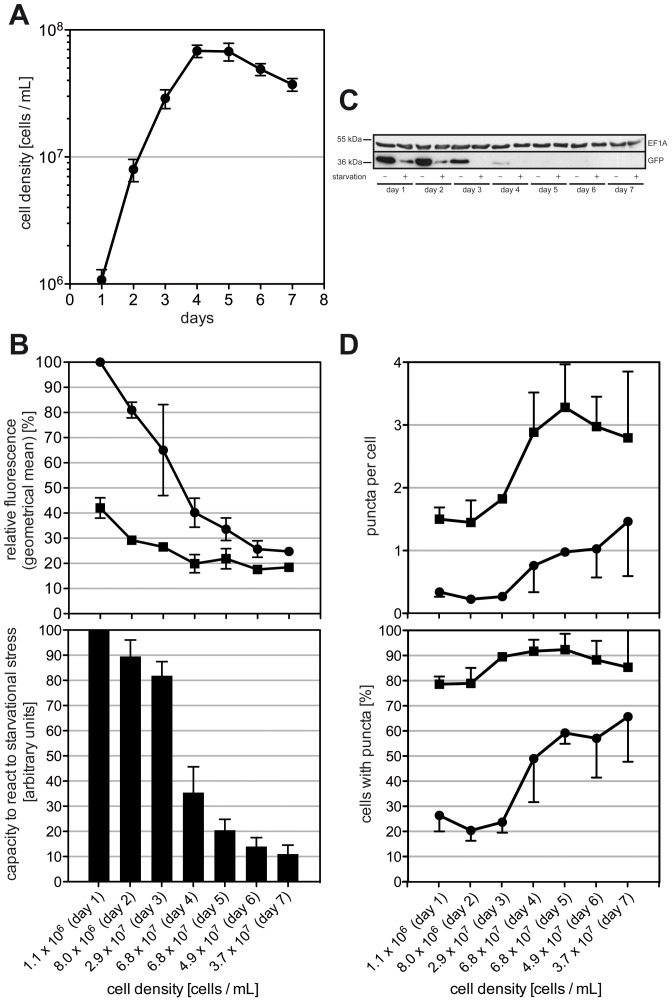
Processing of GFP-TbAtg8 during parasite growth in culture. *T. brucei* procyclic forms expressing GFP-TbAtg8.1 were cultured in mid-log phase for several days, then diluted to 3×10^5^ cells/mL at day 0 and kept in culture without further dilution for 7 days. (A) Parasite density (mean values ± standard deviations from three independent cultures). (B) Each day, GFP-TbAtg8.1 fluorescence was determined by flow cytometry before (•) and after (▪) starvation for 2 hours in gHBSS and expressed relative to the values obtained at day 1 (upper panel). The differences between GFP-TbAtg8.1 fluorescence before and after starvation were plotted to obtain a number for the capacity of parasites to undergo autophagy. The values are expressed relative to the difference observed on day 1. (C) Immunoblot of parasite lysates (5×10^6^ cell equivalents per lane) before (−) and after (+) starvation probed with antibodies against GFP (lower panel) or eukaryotic elongation factor 1A (upper panel, used as loading control). (D) Quantification by fluorescence microscopy of GFP-TbAtg8.1 puncta per cell and cells containing puncta in parasites before (•) and after (▪) starvation. At each time point, at least 100 cells per experimental condition were counted. Error bars indicate standard deviations from three independent experiments.

Autophagy has been shown to affect cell growth in various organisms. In *Arabidopsis*, disruption of autophagy lead to earlier chlorosis under starvation conditions and to earlier leaf senescence under normal conditions [42]. In addition, it was shown to be involved in life-span extension in *Caenorhabditis elegans* [43] and increased longevity in *S. cerevisiae* in the non-dividing state [44]. In the protozoan parasite *Entamoeba invadens*, the number of Atg8-associated structures changed significantly during growth [45]. Based on these reports, we investigated if GFP-TbAtg8.1 puncta formation and processing may also be affected during growth of *T. brucei* procyclic forms under standard culture conditions. Parasites were cultured for 7 consecutive days without dilution (Fig. 8A) and GFP-TbAtg8.1 fluorescence was determined by flow cytometry. The results show that fluorescence intensity decreased with increasing cell density (Fig. 8B, upper panel), indicating that parasites underwent autophagy as the cell density increased. Subsequently, parasites were challenged during late logarithmic and stationary growth phase by starving for 2 hours in gHBSS to determine their ability to cope with additional stress. The results show that starvation led to a further decrease in GFP-TbAtg8.1 fluorescence (Fig. 8B, upper panel), however, the starvation-induced reduction in fluorescence intensity decreased with increasing cell density. Using the difference in signal intensity before and after starvation as parameter to express the capacity/ability of parasites to perform starvation-induced autophagy (Fig. 8C, lower panel) showed that the ability of *T. brucei* procyclic forms to undergo autophagy sharply decreased at day 4, i.e. when parasite cultures reached maximal density and entered stationary phase. A similar change in autophagic behavior was also seen when counting the number of GFP-TbAtg8.1 puncta per cell (Fig. 8D, upper panel) or the number of cells containing puncta (Fig. 8D, lower panel). In parallel, protein levels were analyzed by SDS-PAGE and immunoblotting, showing that GFP-TbAtg8.1 decreased with increasing cell density, and after starvation (Fig. 8C).

## Discussion

In the present report, we demonstrated that autophagy in *T. brucei* procyclic forms can be monitored by flow cytometry using GFP-TbAtg8.1- or GFP-TbAtg8.2-expressing parasites. The decrease in GFP fluorescence intensity strictly correlated with puncta formation, a hallmark of autophagy [9], [40]. In addition, we showed that the number of puncta per cell and the number of cells containing puncta increased during autophagy and was independent of the tag used to label TbAtg8. Our results are in good agreement with previous studies using flow cytometry to monitor autophagy in other organisms [28], [29]. The use of flow cytometry allows rapid determination of autophagic activity, avoiding time-consuming and laborious counting of puncta by fluorescence microscopy or determination of Atg8 levels by SDS-PAGE and immunoblotting. Although the readouts correlated in our study, it should be noted that puncta formation and the degradation of GFP-TbAtg8.1 represent different steps in the process of autophagy. Thus, it is possible that depending on the experimental setup the two readouts may behave differently.

It has previously been shown that autophagy in *T. brucei* procyclic forms induced by starvation in gHBSS can be inhibited by addition of amino acids to the buffer [27]. We now confirmed this finding using flow cytometry to monitor autophagy. In addition, we unexpectedly found that the addition of only a single amino acid, histidine, prevented starvation-induced autophagy in *T. brucei*. No other amino acid, or no other combination of amino acids unless it included histidine, was able to inhibit autophagy-induced decrease in GFP-TbAtg8.1 fluorescence, indicating that prevention of autophagy was not related to energy production from bulk amino acids. At present, we have no clear explanation for this surprising observation.

Studies on the amino acid requirement of autophagy have previously been carried out also in mammalian cells. In Chinese hamster ovary cells stably expressing GFP-LC3, flow cytometry was used to monitor autophagy in a similar way as in our study [28]. As in *T. brucei*, the addition of all twenty proteinogenic amino acids to the starvation buffer prevented autophagy. In a subsequent series of experiments in which one particular amino acid at a time was left out from the incubation, the authors found that the omission of arginine, leucine, lysine or methionine had a major effect on progression of autophagy [28]. Since the design of the experiments was different, i.e. omission of single amino acids from the incubation in their study versus addition of single amino acids to the buffer in our work, a comparison of the results is difficult. In addition, in a more recent study using human embryonic kidney cells stably transfected with GFP-LC3, LC3 processing and puncta formation was induced by deprivation of a single amino acid, leucine, from the incubation buffer [46]. In addition, leucine supplementation inhibited autophagy in a human osteosarcoma cell line expressing Myc-tagged LC3 [46]. These effects were suggested to involve a regulatory role of leucine in autophagosome biogenesis.

In addition, using flow cytometry to measure GFP-TbAtg8.1 fluorescence, we found that autophagy is up-regulated in late logarithmic growth phase, i.e. when *T. brucei* procyclic forms enter stationary phase. Growth arrest of cells in culture may be the result of decreasing availability of nutrients, or quorum sensing mechanism to prevent overgrowth [47], which otherwise may lead to sudden shortage of energy sources. Thus, it may not be surprising that autophagy, which allows the cell to recycle intracellular components to limit nutrient uptake from the medium, is induced during this phase of growth. Our results, however, are in contrast to a study in another protozoan parasite, *Entamoeba invadens*, where autophagy as measured by Atg8 puncta formation was reported to be decreased in stationary growth phase [45]. Finally, using flow cytometry we observed a decreased capacity of parasites to undergo autophagy once they reach stationary phase, which correlates with increased basal level of puncta in cells, and with cells having puncta. Together these data indicate that autophagic flux is increased when *T. brucei* parasites reach higher cell densities and remains at high levels during stationary phase, impairing the parasites' ability to cope with further stress.

## Supporting Information

Figure S1 **Rare co-localization of GFP-TbAtg8.1 with TbCatL.** Experimental conditions are identical as described in the legend to Fig. 3. Co-localization of GFP-Atg8.1 (in green) with TbCatL (in red) is seen in <15% of parasites. DNA is stained with DAPI (in blue); DIC, differential interference contrast.
(TIF)

Table S1 **Primers used for cloning. Restriction sites are underlined; annealing regions are shown in bold print.**
(TIF)

Table S2 **Concentrations of amino acids present in SDM-79 and in the buffer used to prevent autophagy (see** **Fig. 5** **).**
(TIF)
