# Supplementary figures and images for: Autophagy in Trypanosoma brucei: Amino Acid Requirement and Regulation during Different Growth Phases
Source: PLoS One. 2014 Apr 3;9(4):e93875. doi: 10.1371/journal.pone.0093875 (PMC3974859; doi:10.1371/journal.pone.0093875)

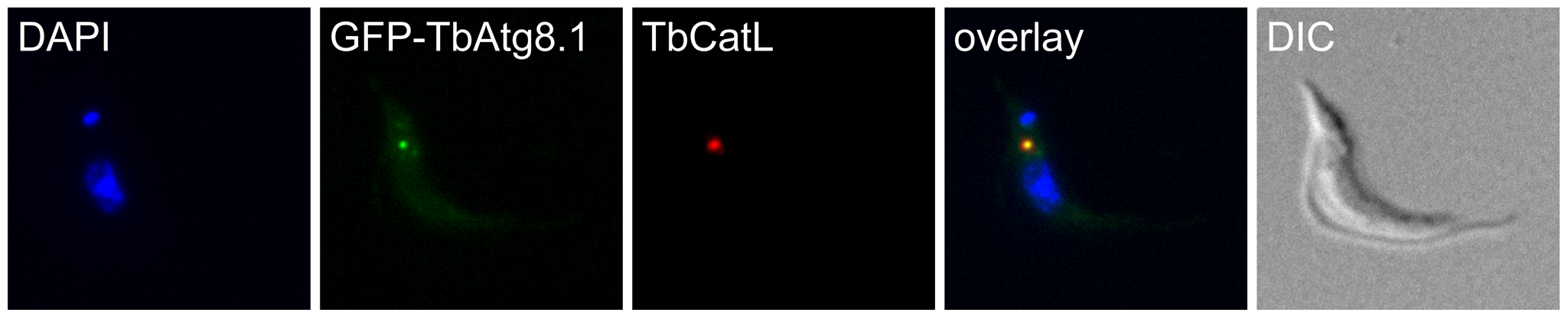

Supplement: Figure S1 — Rare co-localization of GFP-TbAtg8.1 with TbCatL. Experimental conditions are identical as described in the legend to Fig. 3. Co-localization of GFP-Atg8.1 (in green) with TbCatL (in red) is seen in <15% of parasites. DNA is stained with DAPI (in blue); DIC, differential interference contrast. (TIF) [file pone.0093875.s001.tif]

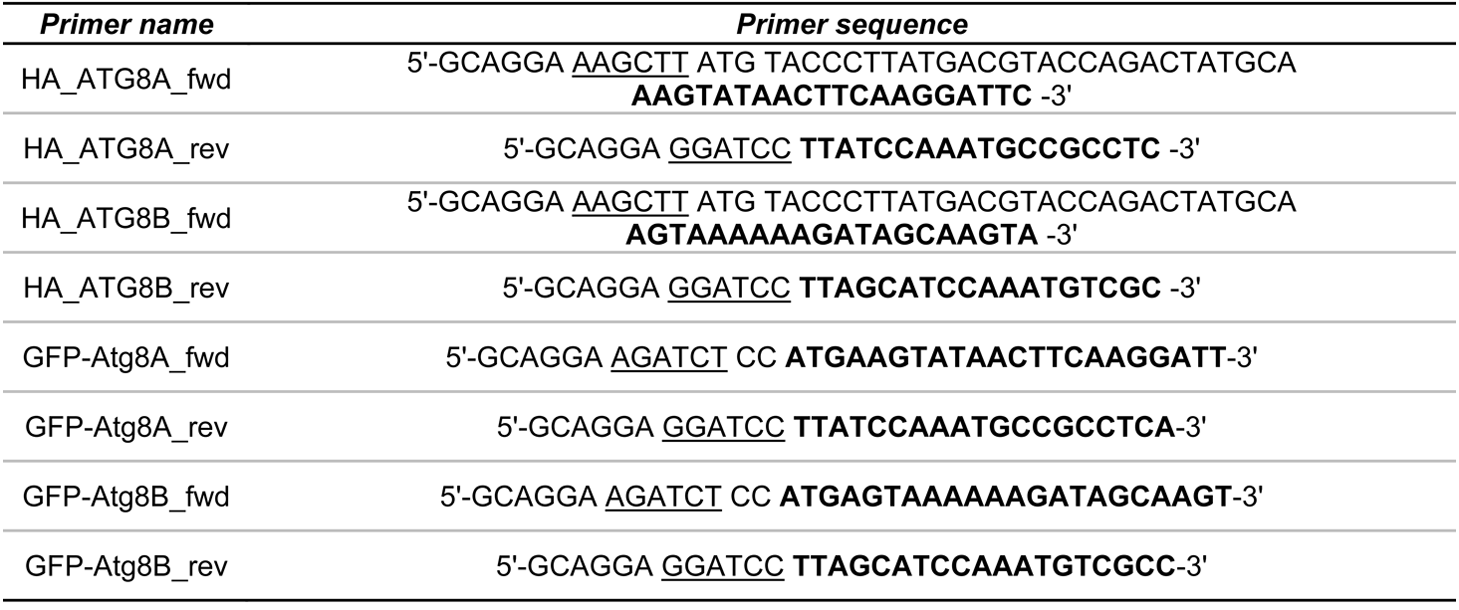

Supplement: Table S1 — Primers used for cloning. Restriction sites are underlined; annealing regions are shown in bold print. (TIF) [file pone.0093875.s002.tif]

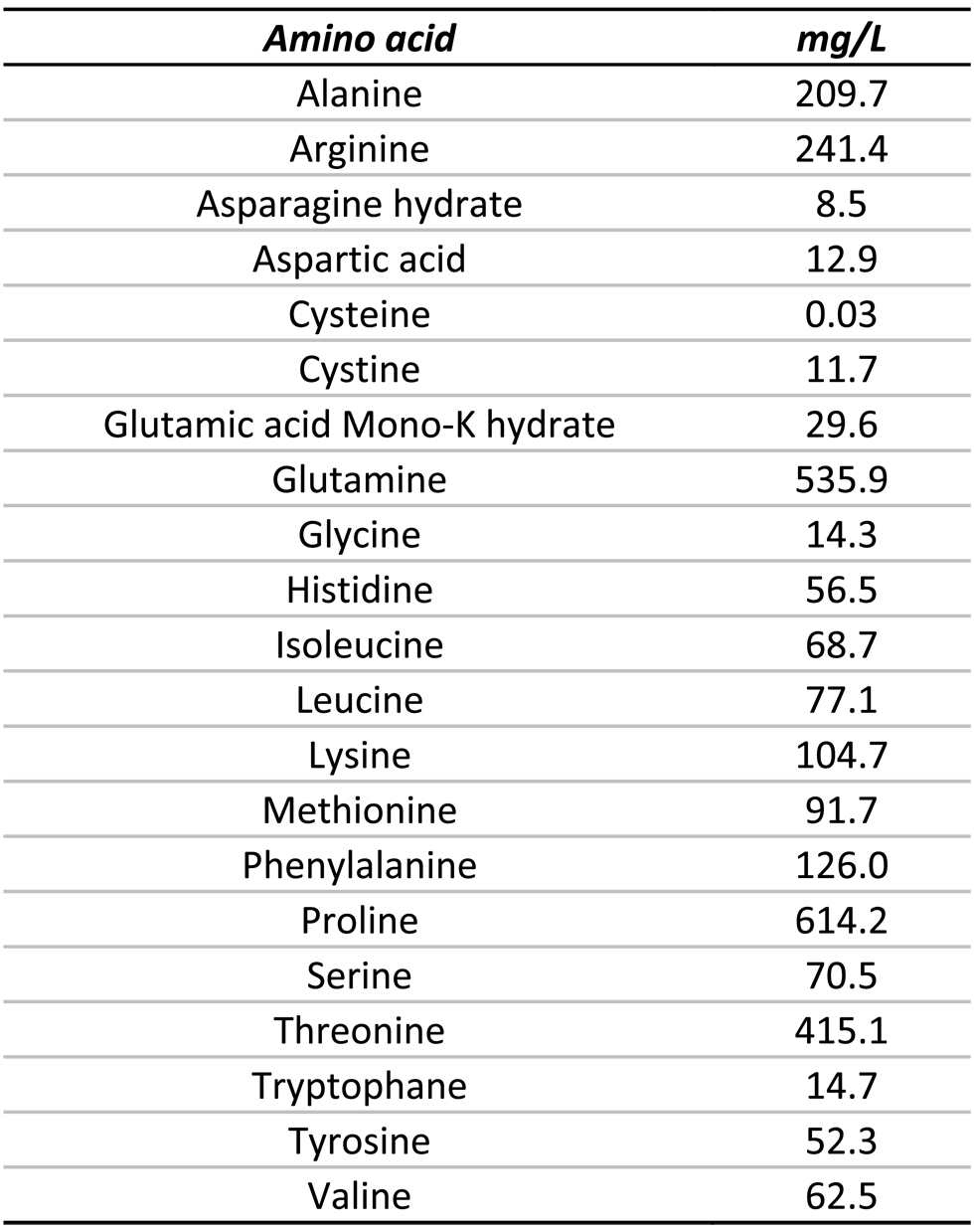

Supplement: Table S2 — Concentrations of amino acids present in SDM-79 and in the buffer used to prevent autophagy (see Fig. 5 ). (TIF) [file pone.0093875.s003.tif]
